# Did COVID-19 change the political communication of polarizing leaders? The case of Salvini's campaigning before and after the pandemic

**DOI:** 10.1177/02673231221140697

**Published:** 2022-11-24

**Authors:** Alberto Bitonti, Rita Marchetti, Claudia Mariotti

**Affiliations:** 27216 Università della Svizzera italiana, Switzerland; Università di Perugia, Italy; 165510 Università degli Studi di Roma Tre, Italy

**Keywords:** Political communication, polarization, campaigning, COVID-19 pandemic, Lega

## Abstract

In recent years, political polarization saw a significant rise in many political systems. This revamped a scientific debate sparked decades ago, with different schools of thought debating on dynamics, factors, and causes of polarization itself. By looking at political elites’ polarizing strategy—one of the factors on which various theories seem to converge—this article tackles the question concerning the impact of the COVID-19 pandemic in terms of political communication. More specifically, we look at the case of a highly polarizing leader in Italy—Matteo Salvini, leader of Lega—in two campaigns held in 2020 before and after the first wave of the pandemic. By analyzing his messages on Facebook and Twitter, we build on the literature on the causes of affective polarization to study Salvini's use of partisan identity and divisive issues, also considering other crucial elements, such as the attacks against others, and followers’ engagement. The results highlight some changes between the two phases, but also a strong continuity in the polarizing strategy of Salvini's political communication.

## Introduction

In recent years, political polarization saw a significant rise in several political systems. Many consider this as one of the great challenges of contemporary democracy, as polarization undermines some of the basic pillars of democracy itself, such as trust and respect for institutions, a shared public sphere, or the valuing of compromises in political processes. The assault on Capitol Hill in the US in January 2021 only made this phenomenon more evident to the public, but the scientific debate on polarization had already been revamped since the end of the last century, becoming increasingly heated and multifaceted.

Various schools of thought have emerged in the last couple of decades, discussing the relationship between elites’ and voters’ polarization (Fiorina and Abrams, 2008; Fiorina et al., 2005), the affective dimension of polarization (Iyengar et al., 2012; Mason, 2015), and the causes of such polarization (Iyengar and Westwood, 2015; Webster and Abramowitz, 2017; West and Iyengar, 2022).

While the behavior of political elites is transversally seen as a crucial factor in determining—as well as reducing—political polarization, several studies have shown that political elites are incentivized to maintain their polarizing strategies (Iyengar and Krupenkin, 2018), using increasingly divisive and hostile rhetoric toward the opponents (transformed into enemies), growing negative feelings toward the others, and being increasingly pushed to more extreme ideological positions, also to gain more visibility in the media and achieve more engagement from the public (Hart et al., 2020; Marozzo and Bessi, 2017; Wagner and Gruszczynski, 2017).

In 2020, the COVID-19 pandemic represented an exceptional test for the political system of many countries, affecting the dynamics of the relationship between majorities and oppositions, and between governments and public opinion. The question this article aims to tackle is: what was the effect of the pandemic on political polarization? More specifically, did COVID-19 change the political communication of polarizing leaders?

In this study, we chose to focus on the Italian context, which in 2020 saw two different rounds of regional elections, one before the first wave of the pandemic (January 2020), and one immediately after (September 2020), thus representing a convenient case study to test the role of COVID-19 in producing specific changes in terms of political communication. Furthermore, we chose to focus in particular on the political communication of Matteo Salvini, leader of the right-wing party Lega, for three reasons: (1) after the results of the political elections of 2018, Salvini had become the leader of the center-right coalition, so that in 2020 he had the highest strategic interest in “winning” the regional elections to politically “harm” the center-left government of the time (Conte II, mainly supported by the Five-Star Movement and by the Democratic Party); (2) he represents the most highly polarizing political leader in Italy, strongly relying on partisanship, divisive issues, and attack-oriented campaigning (Bordignon, 2020; Bosco and Verney, 2020; Mariotti et al., 2021; Rega and Marchetti, 2021; Tronconi and Valbruzzi, 2020); (3) in terms of political communication, he proved to be one of the most effective and tech-savviest campaigners, among the most active and successful political leaders not only in Italy but throughout Europe (looking at his activities online, numbers of followers, levels of engagement, etc.).

The article is structured as follows: we first summarise the debates and the literature on ideological and affective polarization, and on the effects of the pandemic on polarization; in the following sections, we develop some research hypotheses to test in the Italian context and we illustrate our methodology; in the final sections we present and discuss the results as well as the limitations of our study.

## Literature review

### The scientific debate on political polarization

Despite Giovanni Sartori being the initial and main theorist of political polarization (Sartori, 1976, 1982), most of the literature on this topic focuses on the American case. However, referring essentially to the American literature for the concept of polarization would be misleading. First, because the literature on political polarization in Europe (and in the rest of the world) exists and continues to grow (Casal Bértoa and Enyedi, 2022; McCoy et al., 2018), and second, because the US party system can be considered to some extent exceptional for its genuinely bipartisan nature, with some particular aspects that we do not find in multi-party contexts.

In the US, the two-party system has long been accused of not functioning properly, precisely because of the indistinguishable values of the two parties that made it up (Layman et al., 2006), and the same accusation of excessive ideological convergence was leveled at the European mainstream parties since 1980 (Katz and Mair, 1995).

The scenario radically reversed in the US starting from 2000 (Layman et al., 2006), and shortly after in Europe (with the rise of the populist wave), when political elites realised how profitable, in terms of votes, polarization can be, choosing increasingly extreme positions and creating a vicious circle in which the electorate not only became more and more polarized but rewarded polarized elites even more (Jacobson, 2000).

Recently, the debate on political polarization has essentially evolved on the concept of affective polarization (Iyengar et al., 2012), which focuses on the continuous increase of negative emotions against political opponents (considered more and more as an enemy rather than an adversary) present in both the elite and the electorate, and on its causes. A fervent academic debate was generated on the main causes of affective polarization with an identity approach (Iyengar et al., 2012; West and Iyengar, 2022) opposed to the ideological and policy polarization tradition (Jacobson, 2015; Webster and Abramowitz, 2017)^1^.

The branch of study focusing on ideological and policy polarization refers to the ideological distance measured on the classic right-left axis and then moved to divisive policies. It was initially divided between maximalists (Abramowitz, 2010, 2013; Jacobson, 2000, 2015; Webster and Abramowitz, 2017) and minimalists (Fiorina and Abrams, 2008; Fiorina et al., 2005; Levendusky, 2009), where the former argued that the ideological polarization on policies was a phenomenon that pervaded elites and voters, while the latter considered only the elites. Maximalists prevailed over minimalists, even if the debate has recently evolved around the concept of “false” (or perceived) polarization (Levendusky and Malhotra, 2015; Wilson et al., 2020). The perspective of ideological and policy polarization identifies the growing ideological distance between policies as the main cause of affective polarization. According to these scholars, it is thinking so differently on many issues—and not, first of all, partisanship—that generates negative feelings toward opponents and an inability to communicate among the elites (Fiorina and Abrams, 2008; Webster and Abramowitz, 2017).

The identity approach, rooted in studies on collective identity based on experiments in social psychology, argues that any form of group belonging tends to activate two types of emotions: positive in evaluating one's group and negative in evaluating the others’ group (Billig and Tajfel, 1973; Tajfel et al., 1971). Based on these assumptions, the identity approach focuses on partisanship meant as a social identity, considered a crucial factor (at the same level of race or gender) in orienting many choices in everyday life, such as being willing to pay less, for the same job, a person who belongs to the opposing party (McConnell et al., 2018), or in choosing where we would like to work or shop, with whom we would like to go out (Huber and Malhotra, 2017), who to have as a roommate (Shafranek, 2021), to the point of influencing the choice of who to marry (Iyengar and Krupenkin, 2018). Affective polarization, according to this approach, is caused by identifying socially with a party, an identification that generates positive feelings toward the members of one's party (in-group favoritism) and consequently feelings of hostility toward those who do not belong to it (out-group animus).

In a recent experiment, some exponents of the identity approach (West and Iyengar, 2022) confirmed the importance of partisanship but failed to prove the causal relationship between it and negative feelings toward the other. They conclude that affective polarization may be caused by several factors, beyond partisanship, such as the ideological polarization on policies, the role of social media in providing user-friendly information (Peterson and Iyengar, 2021), and the use of violent and uncivil rhetoric used by political elites in “negative” campaigns (Geer, 2010; Kenski et al., 2018). Building on this debate on the causes of affective polarization, we analyze Salvini's campaigning polarization through the categories of partisan identity and divisive issues—also considering the attacks against others, and followers’ engagement.

### Polarization and COVID-19

In 2020, the COVID-19 pandemic shook the political systems all over the world, also affecting the dynamics of political polarization.

The first pieces of research on this aspect have focused in particular on the 2020 US presidential campaign. Gollust et al. (2020) have defined the pandemic crisis not only as a health crisis but also as a political communication one, placing a clear responsibility on the communication of political elites, who, especially in the first months of the diffusion of the virus, have conveyed not only discordant but even opposite messages, spreading great confusion among the public, resulting in the consequence that the public's attitudes were driven above all by affective polarization, rather than by the search for reliable information (Green et al., 2020). Nevertheless, the subsequent short convergence in the communication of the opposing US political elites on the management of the health emergency (started around April 2020) led to a convergence in the behavior of the voters as well, mitigating the influence of affective polarization (Gollust et al., 2020; Green et al., 2020). Other recent studies (Druckman et al., 2020) have focused on the role played by the pandemic in mitigating the ideological polarization on policy preferences of the electorate, showing that in facing an emergency context, undeniable in its dramatic nature, the choice to support a policy is influenced less than usual by the political alignment, demonstrating consequently that affective polarization can be reduced.

However, even considering the potential mitigating (or null) impact of the pandemic (in its first stage) on political polarization, it is clear that a decrease in polarization would only be possible if the political elites put aside or at least decreased their violent and divisive rhetoric (Grossman et al., 2020; Jungkunz, 2021), and if the parties decided to favor the choice of more moderate candidates (Lelkes, 2018).

In Europe, research has focused on the link between populist parties^2^ and COVID-19, showing that the politicization of the pandemic has been quite difficult for most of them, especially in the first phase of the pandemic (Bobba and Hubé, 2021). These studies show that European populist parties very soon agreed on the gravity of the emergency led by the pandemic, supporting special containment measures, intensifying—for the right-wing parties—the emphasis on nationalism and the polarizing discourse based on “we, the national people,” not only against the European Union but also against the other EU Member States. European populist parties started to differ in their campaign strategies more in the last phase of the pandemic, when its gravity was somehow weakening. Most of them got back to their classical polarizing issues, updating their discursive repertoire to the new COVID-19 age, with criticism against a slow and uncoordinated EU-elite's responsiveness as well as against migrants (receiving too much attention from the government in comparison to domestic citizens, or portrayed as vehicles for the virus). Others opted for a radicalization of the political discourse, fostering more intense campaigns against the “enemies of the people” (Bobba and Hubé, 2021; Casal Bértoa and Enyedi, 2022).

## Research hypotheses

Taking Matteo Salvini as a typical (and quite relevant) case of a political leader strongly relying on polarization in his communication strategy, and considering the literature illustrated above, our study aims at investigating whether and how the COVID-19 pandemic affected Salvini's political campaigning in terms of communication strategy.

Focusing on the two rounds of campaigns for the Italian regional elections of 2020, with the former held just before the pandemic (January 2020) and the latter immediately after the end of the first wave (September 2020), we analyzed the corpus of Matteo Salvini's tweets and Facebook posts, aiming to empirically test some expectations.

As seen in the previous section, the two major schools of thought on polarization identify divisive policies and partisan identity as the main causes of affective polarization. We already know that both categories are crucial in Salvini's communication (Mariotti et al., 2021), therefore, the goal of this study is to see whether and how the COVID-19 pandemic changed the role played by these categories in Salvini's campaigning.

Considering the literature on COVID-19 and right-wing populist parties’ communication in European countries, we expect that, after the first wave of the pandemic (when the emergency had somehow declined), Salvini may have tried to gain public support by exploiting traditional divisive issues such as immigration, security, and euro-criticism, re-framing these issues in a COVID perspective (e.g., portraying immigrants as vehicles of the virus); at the same time, we expect Salvini's communication to focus on new divisive issues emerging from the crisis, such as the management of the pandemic by the government and the various policies connected to it (relief packages, issues in education and employment, etc.). Therefore, our first hypothesis is:

H1. When the emergency of COVID-19 declines, polarizing leaders get back to exploiting traditional divisive issues, re-framing these issues in a COVID-19 perspective.

We already know that partisan identity (operationalized as references to the right/left ideological axis and national identity) is a crucial content in Salvini's posts and tweets, and one of those that receive the highest level of engagement (Mariotti et al., 2021). However, different expectations might be drawn on how the pandemic affects these two components of partisanship. Taking into account that a common enemy (natural calamities, wars, pandemics) can foster a strong sense of national unity (Achen and Bartels, 2017; Lenz, 2012), we expect that references to the right/left ideological axis decrease, while references to the national identity increase.

H2: The perception of COVID-19 as a common enemy leads polarizing leaders to interpret partisan identity more in terms of national identity than right/left axis.

The literature on polarization indicates the attack-oriented, divisive, and violent rhetoric conveyed by political elites as a crucial cause of affective polarization (Druckman et al., 2020). Although this type of rhetoric has been employed mainly in the US (Lau and Rovner, 2009), the negative strategy has more recently affected the campaigning of European parties and candidates (Nai and Sciarini, 2018; Usherwood and Wright, 2017). Various factors—such as candidate-centered campaigning strategies and the personal management of social media by political leaders—determined an increased use of attacks (Krupnikov and Piston, 2015).

The literature in this field shows that attack messages often tend to become uncivil. Especially on digital platforms, the use of uncivil language has the dual function of (1) strengthening the identity of the group, also facilitating socialization and possibly the recruitment into one's group and (2) increasing the negative feelings against the opposing group (Bentivegna and Rega, 2020; Muddiman, 2019). In this perspective, identity is mainly linked to the networks whose one is a member and can evolve as political or party identity.

As highlighted by previous studies (Berti and Loner, 2021; Mariotti et al., 2021; Rega and Marchetti, 2021), Salvini makes abundant use of attack-oriented messages.

In line with the recent literature on European populist right-wing parties’ politicization of the pandemic, we expect an increased recourse to attack-oriented and uncivil messages in Salvini's communication, targeting, above all, the government and its management of the crisis.

Our third hypothesis then is:

H3. After the first wave of COVID-19, polarizing leaders increase the use of attack-oriented and uncivil messages, above all against the governmental management of the crisis.

Studies on affective polarization highlight how it arises from a vicious circle (Jacobson, 2000; Lenz, 2012), where elites not only tend to exacerbate the negative feelings toward the opponents and their policies (consequently activating the electorate's affective polarization), but voters themselves demand a hard and uncompromising line against the opponent/enemy from the elites. In other words, the messages of political leaders that favor divisive issues and incivility exert a sort of “dark attraction” on users (Rega and Marchetti, 2021: 125).

We know that Salvini's messages concerning divisive issues and references to partisan identity (two of the main causes of affective polarization) received a high engagement in the past (Mariotti et al., 2021). Besides, if—as expected in H1—with the decline of the emergency Salvini chooses to get back to a polarizing strategy (like other European right-wing populist parties), it would make sense to observe a high engagement on (new and old) divisive issues as well as on partisan identity (in line with the previous elections).

Our fourth hypothesis then is:

H4. When the emergency of COVID-19 declines, divisive issues and partisan identity, especially if uncivil, receive a high level of engagement, in continuity with the pre-COVID-19 times.

## Data and methodology

To test the research hypotheses, the analysis focuses on posts and tweets published by Salvini on his Facebook official page (@salviniofficial) and Twitter account (@matteosalvinimi), during the two campaigns for the Italian regional elections of 2020^3^, held before and after the first wave of the pandemic. The Facebook posts were downloaded using CrowdTangle, the tweets through Vicinitas. We chose Facebook and Twitter for different reasons: Salvini is widely recognized for his massive use of social media (Bobba, 2019), with Facebook specifically playing a primary role, as he is followed by more than 4 million people (Table 1). Also, Facebook is the platform with the highest number of users in Italy,^4^ and political discussion on social media is a significant part of citizens’ participation in public life. Twitter, instead, is a relevant arena for political communication, impacting not only the electoral process but also influencing news coverage and agenda-setting, with a user base stable over time ranging from politicians and journalists to academics, celebrities, etc. Table 1 shows the number of posts/tweets analyzed and some descriptive statistics.

**Table 1.** Dataset summary.

	Before the pandemic (January 2020)		After the end of the first wave of the pandemic (September 2020)	
	Facebook	Twitter	Facebook	Twitter
Number of posts/tweets	1560	2397	1637	3103
Received total interactions (average)*	20,203	1863	29,539	1140
Number of followers**	4,041,900	1,368,222	4,819,946	1,368,220

*In the Facebook case, the received total interactions are the sum of comments, shares, and other reactions (love, wow, haha, sad, angry, and care). In the Twitter case, the received total interactions are the sum of favorites, retweets, replies, and quotes.

**The data were collected respectively on January 26, 2020, and on September 21, 2020.

To identify the content of posts/tweets in terms of functions, types of issues, and presence of attacks, all the messages have been analyzed through QDA Miner, a software for the qualitative analysis of texts, and its quantitative component, WordStat, a text-mining tool used to identify recurring themes in a text.^5^ After a first explorative analysis (*topic model*), useful to identify the categories of analysis, the texts were classified using a supervised machine learning algorithm, identifying one or more predefined categories based on a process of inductive learning, trained on a set of texts previously classified manually. Specifically, two “search and retrieval” tools were used: “Query by example” and “Code similarity”. At the end of the analysis, two different researchers classified a random sample representing 5% of the corpus (160 posts and 275 tweets), assigning the codes listed in the codebook.^6^

We identified the function of each content published by Salvini on the two platforms comparing the data on the first campaign (before the pandemic) and the second campaign (at the end of the first wave). As shown in Table 2, the statements on political and campaign themes (*stances on political issues*) have grown in the second campaign both on Facebook and Twitter (respectively by 15 and 17 percentage points), while other types of content—information on meetings and other activities in the territory (*campaign updates*), calls to action to supporters (*mobilization*), content related to family, daily life, or food (*intimacy*), and the relaunch of interviews and clips from other media (*self-promotion*)—decreased. The decrease in messages of campaign updates and mobilization is most likely due to the restrictions imposed by the pandemic-containment rules, strongly reducing the possibility to organize rallies and local political events.

**Table 2.** Functions of Facebook posts and tweets.*

	Before the pandemic (January 2020)		After the end of the first wave of the pandemic (September 2020)	
	Facebook	Twitter	Facebook	Twitter
Stances on political issues	40.8	37.3	55.9	54.0
Campaign updates	30.6	23.4	29.0	19.0
Mobilization	22.9	13.5	14.1	8.5
Intimacy	20.5	17.3	17.1	12.6
Self-promotion	8.7	13.9	8.2	8.5
Total	(1560)	(2397)	(1637)	(3103)

* Within each post/tweet it was possible to record more than one function. Percentages and totals are based on the total number of posts/tweets analyzed.

To test H1 and H2, this analysis was complemented by a deeper content analysis concerning Salvini's stances on political issues, to assess his resort to divisive issues and references to partisan identity, two of the main causes of affective polarization (see the literature review above).

Partisan identity refers to messages concerning the traditional political *right-left ideological axis* and *national identity*. National identity^7^ has been chosen as an indicator of partisan identity for two reasons: first, it is a characteristic of the identity of right-wing parties, in particular Salvini's Lega; second, it is considered one of the main identity dimensions underlying in-group bias (Druckman, 1994; Tajfel, 1982).

The other stances on political issues have been split into *divisive issues*^8^ (immigration, security, COVID-19, education, employment, Europe, taxes, Bibbiano case,^9^ local maladministration operated by opponent political parties, costs of politics, social welfare) and *nondivisive issues* (economic development, other) (Tronconi and Valbruzzi, 2020).

To test H3, we detected all the cases of attacks toward the opponents and the presence of incivility. The attacks (operationalized as messages portraying opponents negatively), were categorized as *attacks against the elites* (mainly political, but also media and economic elites) and *attacks against “others”* (Islam, immigrants).

The presence of incivility was instead detected distinguishing *impoliteness* (violation of the rules of courtesy, teasing, sarcasm) and *hate speech* (discrimination, inciting hatred or violence toward people with given traits or attributes) (Rega and Marchetti, 2021).

To detect the two forms of incivility present in the corpus under examination, through the WordStat tool, an already available dictionary of uncivil words and phrases was used (Rega and Marchetti, 2021), integrated with words or phrases previously not included in the dictionary and specifications of the communication strategy used by Salvini in the two rounds of campaigns considered.

Finally, to test H4, we analyzed users’ interactions concerning the content published by Salvini on the two platforms.

## Results

The analysis of Salvini's stances on political issues in the two campaigns (Table 3) shows an increase in messages concerning divisive issues in the second campaign. The percentage of cases containing divisive issues increases over time, ranging from 64.5% before the pandemic to 78.8% after the end of the first wave of the pandemic on Facebook, and from 67.6% to 86.7% on Twitter. The immigration issue increased the most (doubling its presence on Facebook in the second campaign, and reaching 46.2% of cases), being re-framed in a COVID-19 perspective by presenting immigrants as a vehicle of the virus, as emerging from the examples reported below, providing support for our first hypothesis (H1).

… Police officers are forced to work to contain positive illegal immigrants refusing hospitalization. Gowns for Law-Enforcement officers, fines and restrictions for Italians but open harbors for infected immigrants roaming throughout Italy [Facebook post, 18^th^ August 2020]

**Table 3.** Stances on political issues (%).

		Before the pandemic (January 2020)		After the end of the first wave of the pandemic (September 2020)	
		Facebook	Twitter	Facebook	Twitter
Partisan identity	National identity	24.5	20.4	10.3	6.6
	Right/Left ideological axis	10.3	4.0	4.0	3.0
	*Subtotal*	*34.5*	*24.3*	*13.2*	*9.5*
Divisive issues	Immigration	22.7	20.9	46.2	31.9
	Security	18.9	18.2	21.9	15.5
	COVID-19	—	—	21.2	16.7
	Education	—	—	11.9	14.7
	Employment	—	—	11.1	8.9
	ESM/Europe	10.0	13.7	5.5	4.5
	Taxes	9.0	8.5	6.6	7.8
	Bibbiano case	7.5	8.5	—	—
	Local Maladministration	3.0	1.9	1.6	2.1
	Cost of politics	—	—	1.7	1.6
	Social Welfare	—	—	1.0	1.3
	*Subtotal*	*64.5*	*67.6*	*78.8*	*86.7*
Non divisive issues	Economic development	9.1	9.9	4.3	3.8
	Other	3.8	3.0	2.4	3.0
	*Subtotal*	*11.9*	*12.9*	*6.5*	*6.7*
Total		(603)	(886)	(915)	(1675)

Notes: Within each post/tweet it was possible to record more than one stance on different issues. Percentages and total based on the total number of posts/tweets containing stances on political issues.

Tens of infected among the more than 500 guests, nearly all immigrants, in the facilities of “Missione Speranza e Carità”, in Palermo [tweet, 19^th^ September 2020]

At the same time, new divisive issues arise in Salvini's second campaign (Table 3): 21.2% of posts and 16.7% of tweets concern the pandemic in general, followed by the government policies in the fields of education (11.9% and 14.7%) and employment (11.1% and 8.9%).

The government changes its mind once again and goes back to CLOSE shops and bars, jeopardizing thousands of jobs, while the virus gets imported from abroad [Facebook post, 16^th^ August 2020]

Does it look normal to you that the government gave a bonus for electric scooters, buys desks with wheels, and did not give a euro to schools to buy fever checking devices… [tweet, 26^th^ August 2020]

On the other hand, the references to Europe decrease in the second campaign, whereas before the pandemic there were many EU-related contents (such as on the European Stability Mechanism), framed in strong opposition to the national government. It is reasonable to interpret this strategic choice as related to the context created by the EU decision (specifically in the summer of 2020, during the second campaign) to launch a massive stimulus package of hundreds of billions of euros—the NextGenerationEU (NGEU) Plan—to help the member states dealing with the consequences of the pandemic, a context of great popularity for the European Union, especially in the country (Italy) that would benefit the most from the plan. This would also explain a decrease in the messages concerning the national identity (respectively from 24.5% and 20.4% to 10.3% and 6.6% on Facebook and Twitter in the two campaigns). In sum, the image of “Europe” represented as an enemy of Italy and its excellence results rather resized in the second campaign.

Moreover, the references to the right/left ideological axis decrease as well, on both platforms. This means that H2 is only partially confirmed by the data, as the messages of partisan identity in Salvini's second campaign decrease in both dimensions (Table 3).

Regarding the use of attack-oriented messages (H3), Table 4 confirms that in the second campaign Salvini increases his attacks on the elites, in particular on the government (blamed for its management of the crisis), while decreasing his attacks on others. Even when focusing his messages on immigrants (as already mentioned, the most frequent issue in the second campaign), the target of the attack is usually the government and its strategy to manage migratory phenomena during the pandemic, rather than immigrants themselves.

This government endangers Italy: “ferocious” and severe controls for Italians, while they let hundreds of illegal immigrants, potentially infected, flee from centers. [Facebook post, 27^th^ July 2020]

**Table 4.** Presence of attack and incivility.

		Before the pandemic (January 2020)		After the end of the first wave of the pandemic (September 2020)	
		Facebook	Twitter	Facebook	Twitter
Attack					
	Attack against the elites	32.2	28.8	53.1	41.9
	Attack against “others”	24.9	22.6	17.5	4.5
	Contains no attack	42.9	48.6	40.2	55.9
Incivility					
	Civil	63.8	89.6	73.4	81.8
	Impolite	29.9	9.2	23.0	15.9
	Hate speech	6.3	1.2	3.6	2.3
Total		100.0	100.0	100.0	100.0
		(603)	(886)	(915)	(1675)

Note: Percentages and total based on the total number of posts/tweets containing position statements on issues.

They want to put masks on our children in classes, in the meantime, they let thousands of illegal immigrants and hundreds of infected disembark.

[Facebook post, 24^th^ August 2020]

**Figure 1.**
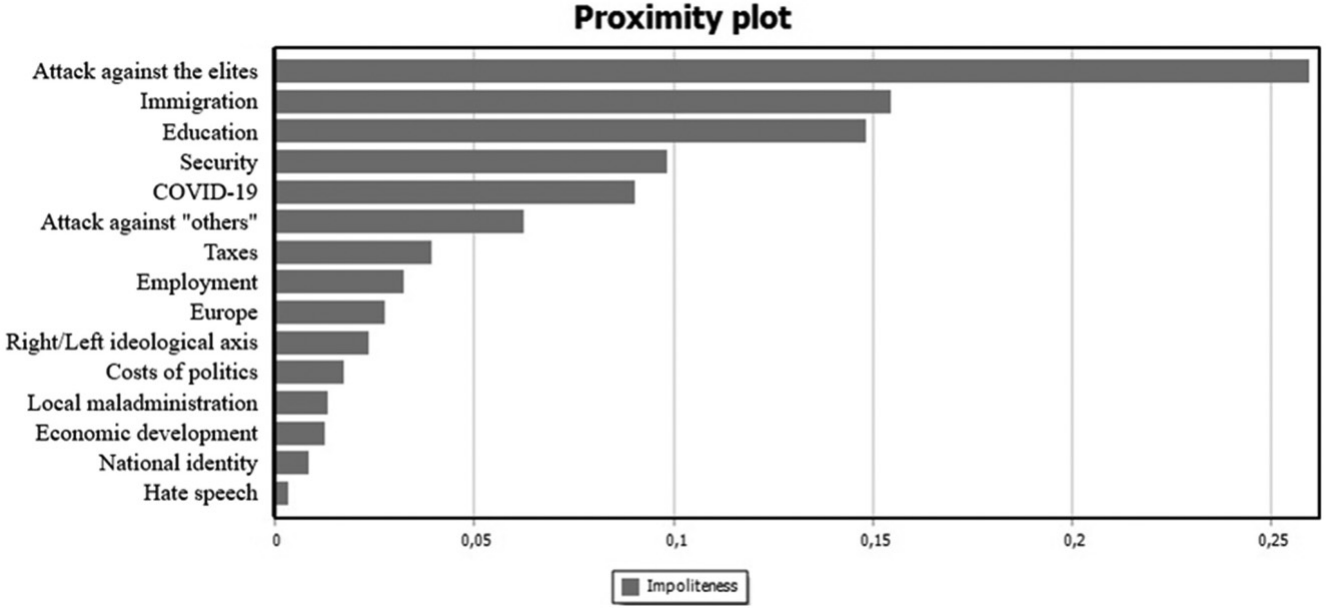
Contents of tweets with the presence of impoliteness (Jaccard Index)^12^ (September 2020).

However, H3 is only partially confirmed, as in the second campaign both types of incivility (impoliteness and hate speech) decrease on Facebook, while impolite messages increase on Twitter, co-occurring with the attacks against the elites, especially when it comes to immigration and education (Figure 1).

The difference in strategy on the two platforms might be explained by the affordances of Twitter as exemplified by the large use of hashtags to attack the government (e.g., #governoclandestino), and in particular the Minister of Education Lucia Azzolina (#azzolinabocciata).^10^

The choice of decreasing uncivil messages on Facebook (even if not in line with the literature on polarizing leaders) can be explained by the peculiarity of the Italian case. In fact, it is necessary to consider: (1) the sense of national unity fostered by the pandemic; (2) the high rates of approval for the Italian government in that period; (3) the fact that Salvini had been decreasing his level of incivility over time in previous elections (Mariotti et al., 2021; Rega and Marchetti, 2021).

We turn now to the relationship between Salvini's posts/tweets and users’ answer in terms of engagement, looking at which types of content are more frequently associated with low, medium, and high classes of engagement (Table 5). We report here only the results specifically related to the engagement of posts on Facebook, as the results on Twitter are almost the same. The findings partially confirm the last hypothesis of this study (H4), namely that after the first wave of COVID-19, Salvini's posts/tweets on divisive issues and partisan identity, especially if uncivil, receive a high level of engagement, in continuity with the previous elections. Differently from what we expected, the messages concerning national identity and Europe received a lower level of engagement, probably due to the high popularity of the EU in helping Italy dealing with the pandemic.

**Table 5.** Engagement classes of different contents of Facebook posts (deviation table)^11^

	Low	Medium	High
Before the pandemic (January 2020)	Self-promotion	Attack against elites	Security
	Taxes	Economic development	Right/left ideological axis
	Mobilization	Local maladministration	National identity
	Campaign updates		Intimization
	Bibbiano case		Europe
			Attack against “others”
			Immigration
			Hate speech
			Impoliteness
After the end of the first wave of the pandemic (September 2020)	Economic development	Education	Security
	Employment	Social Welfare	COVID-19
	Taxes	Europe	Attack against “others”
	Campaign updates	National identity	Hate speech
	Self-promotion		Immigration
	Mobilization		Attack against the elites
	Costs of politics		Impoliteness
	Local maladministration		Right/left ideological axis
			Intimization

In the higher engagement class, we find divisive issues such as security, COVID-19, and immigration, along with hate speech and impolite messages. Also the typically populist messages—focusing on the opposition between “us” (right/left ideological axis) versus “them” (attack against “others”)—receive a high level of engagement, confirming the theories that not only the elites exacerbate the negative feelings toward the opponents, but voters themselves reward a hard and uncompromising line against the opponent/enemy (Jacobson, 2000).

## Conclusions

This paper aimed at contributing to the debate on polarization. Our results seem to be in line with the most recent literature, highlighting the crucial role played by both divisive policies and partisanship (beyond other factors) in fostering affective polarization.

However, this study suffers from some limitations. Although Salvini has been considered an emblematic case of polarizing leader in Italy and Europe, broader samples of political leaders and comparative analyses of multiple contexts would certainly help to draw safer conclusions on the impact of COVID-19 on political communication. Future research might also tackle the communication of other political leaders who are not being characterized as polarizing, looking at their use of partisanship and divisive issues in a comparative framework.

Despite these limitations, the case-study analyzed in this paper confirms a trend emerged in recent research on polarizing leaders, showing that after the pandemic Salvini's communication becomes even more polarizing than before, with a consistent rise of divisive issues and attack-oriented messages.

Specifically, in order to interpret our results, it is necessary to take into account that polarization is not only a political strategy successfully (until these elections) pursued by Salvini, but it also represents a traditional political core of Lega, usually exploited in order to strengthen partisanship as social identity. The communication of Lega has always been characterized by polarizing attack-oriented content, above all when the party has been in the opposition. After the first wave of COVID-19, the situation for Salvini's Lega was problematic: on one side opinion polls were signaling a declining support for the party, while on the other the government (opposed by Salvini) was enjoying large approval ratings. The pandemic, considered as a common enemy that fosters a sense of unity in the people, prevented Salvini from using its traditional polarizing communication for a while, but as soon as the gravity of the pandemic seemed to decrease, Salvini revamped the traditional communication “roots” of his party, hoping to gain electoral support back as already happened in the past.

However, after the first wave of COVID-19, two main differences emerge in Salvini's political communication strategy in comparison with the past. Salvini's usual “designated enemy,” the EU, receives lower attention as a divisive issue and is also attacked less: this change can be reasonably explained in consideration of the salience of the NGEU Plan, the EU massive stimulus package destined to help Italy and other member-states in their recovery from the COVID-19 crisis, that somehow prevented Salvini from relying on this traditional pillar of his polarizing campaigns. The increasing popularity of the EU in Italy (the member-state that would benefit the most from the plan) is most likely behind also Salvini's decreasing references to national identity, whereas this was a crucial content in previous elections. The pandemic fostered a strong sense of national unity, shifting the salience of political differences and transforming the traditional scheme of in-group party against out-group party into a new one based on “us,” a superordinate in-group identity (including Europe, not only Italy), against the virus.

## Notes

1. As early as 1976, Sartori somehow anticipated this debate, dividing the concept of ideology into two different sub-categories: ideological distance and ideological intensity. “The term is used here first to denote an ideological distance, that is, the overall spread of the ideological spectrum of any given polity, and second to denote ideological intensity, that is, the temperature or the affect of a given ideological setting” (Sartori, 1976: 111). Here Sartori refers to partisanship, linking it to the voter's affective perception (rather than actual knowledge) of party policies (Sartori, 1982: 46–47).

2. Populism and polarization are connected concepts as far as we agree that populism always triggers polarization (Mariotti, 2022).

3. In January 2020, regional elections were held in Calabria and Emilia-Romagna; in September 2020, in other 7 regions: Toscana, Marche, Campania, Puglia, Veneto, Liguria and Valle d’Aosta. We analyzed posts/tweets published during the periods from the day of the official submission of candidates’ lists to the day of the vote (respectively from 14^th^ November 2019 to 26^th^ January 2020, and from 22^nd^ June to 21^st^ September 2020).

4. Source: https://wearesocial.com/it/blog/2021/01/digital-2021-italia/.

5. In the case of posts/tweets containing photos, videos, or links, in addition to the text of the message, the text shown on the images (image text), the URL, or the title of the shared video were analyzed.

6. The reliability test gave satisfactory results for all the variables considered (function/position-taking: Krippendorff's *α* = .82; presence of attack: Krippendorff's *α* = .80).

7. This category includes all posts/tweets in which Salvini enhances Italian excellences in terms of culture, sport, made in Italy, but also as Christian identity and roots.

8. Divisive issues here are considered as such in relation to the specificity of the electoral context, as the same definition of divisive issues must take into account the agenda of mainstream media and social media at the time data are collected. For instance, healthcare or gun control can be currently considered divisive issues in the US context, whereas they are not in most European countries (Chen and Berger, 2013; Rega and Marchetti, 2021; see also The Polarization Index report at https://thepolarizationindex.com/).

9. The Bibbiano case was a scandal involving the center-left administration (later acquitted) of the town of Bibbiano (located in Emilia-Romagna, one of two regions involved in the elections in January 2020).

10. In Italian ‘bocciata’ is the term used when failing an exam or a test, while the idea of a ‘clandestine government’ uses an analogy between the status of illegal migrants and that of the cabinet.

11. The analysis shows the ranks of frequencies of contents in each class of engagement. Engagement classes have been calculated considering total interactions. The classes of engagement are respectively distributed into “up to 10,000”/“from 10,001 to 20,000”/“Over 20,000” before the pandemic and “up to 16,000”/“from 16,001 to 30,000”/“Over 30,000” after the end of the first wave of the pandemic.

12. Jaccard coefficient: attack against the elites .259; Immigration .154; Education .148; Security .098; COVID-19 .090; attack against “others” .062; Taxes .039; Employment .032; Europe .027; Right/Left ideological axis .023; Costs of politics .017; Local maladministration .013; Economic development .012.
